# The prospective association between frequency of contact with friends and relatives and quality of life in older adults from Central and Eastern Europe

**DOI:** 10.1007/s00127-020-01834-8

**Published:** 2020-02-10

**Authors:** Eliazar Luna, Milagros Ruiz, Sofia Malyutina, Anastasiya Titarenko, Magdalena Kozela, Andrzej Pająk, Ruzena Kubinova, Martin Bobak

**Affiliations:** 1 Research Department of Epidemiology and Public Health, University College London, 1-19 Torrington Place, London, WC1E 6BT UK; 2 Faculty of Physical Education and Sport, Charles University, José Martího 31162 52, Prague, Czech Republic; 3 Laboratory of Ethipathogenesis and Clinics of Internal Diseases, Institute of Internal and Preventive Medicine, 175/1, Borisa Bogatkova street, Novosibirsk, Russia; 4 Institute of Public Health, Faculty of Health Sciences, Jagiellonian University Medical College, Grzegórzecka 20, 31-531 Krakow, Poland; 5 Centre for Environmental Health Monitoring, National Institute of Public Health, Srobarova 48, 10042 Prague, Czech Republic

**Keywords:** Ageing, Quality of life, Social networks, Depression, Central and Eastern Europe, Older ages

## Abstract

**Purpose:**

Studies suggest that frequent contact with friends and relatives promote mental wellbeing in later life, but most evidence comes from Western populations. We investigated the prospective relationship between frequency of contact with friends and relatives and quality of life (QoL) among older Central and Eastern European (CEE) adults and whether depressive symptoms mediated the hypothesised longitudinal relationship.

**Methods:**

Data from 6106 participants from the Health, Alcohol and Psychosocial factors In Eastern Europe (HAPIEE) study were used. Frequency of contact with friends and relatives was measured at baseline. QoL, at baseline and follow-up, was measured by the Control, Autonomy, Self-realisation, and Pleasure (CASP) 12-item scale. After assessing the prospective association using multivariable linear regression, the mediational hypothesis was tested using path analysis.

**Results:**

There was a significant prospective association between frequency of contact with friends and relatives and CASP-12 score (0–36) in fully adjusted models. Per every one unit increase in frequency of contact, there was a 0.12 (95% CI 0.06, 0.17) increase in CASP-12 score at follow-up, accounting for sociodemographic, health-related and baseline QoL. Pathway results showed that 81% of the longitudinal effect of frequency of contact on QoL was mediated through depressive symptoms.

**Conclusions:**

Frequent contact with friends and relatives improves QoL of older Central and Eastern European adults, partly through buffering against depressive symptoms. Interventions to improve QoL at older ages should incorporate effective management of common mental disorders such as depression.

**Electronic supplementary material:**

The online version of this article (10.1007/s00127-020-01834-8) contains supplementary material, which is available to authorized users.

## Introduction

Eudemonic wellbeing, defined as the fulfilment of one’s potential and purpose in life [1], is an important component of mental health that is crucial for successful ageing. Markers of eudemonic wellbeing have been prospectively linked to lower rates of mortality [2, 3] and morbidity [4–6]. Given the importance of eudemonic wellbeing in later life, ageing experts have sought to understand the key drivers of wellbeing at older ages. Quality of life (QoL) is an important aspect of eudemonic wellbeing and generally refers to a person’s perception of their position in life regarding his or her context and goals [7]. Evidence suggests a decline in QoL when people reach 75 years of age [8]. This decline has been linked to key age-related circumstances, ranging from declining states of physical health, retirement, widowhood and diminished social networks (SN) [8].

Growing interest in the link between SN and QoL has emerged in recent decades. Cross-sectional evidence has suggested that quality, density and frequency of contact with one’s SN predicted greater QoL in older age [9, 10]. More recently, longitudinal evidence has suggested that people with smaller SN have a lower QoL and a faster decline of it at older ages [11]. Analysing age-related changes in wellbeing in different cohorts, Jivraj et al. found that individual differences in the rate of change could be partially explained by the number of close friends and family [8]. Webb et al., found that frequent contact with friends, but not with relatives, predicted higher QoL at follow-up [12]. This factor has been regularly associated with higher QoL among older people in other studies [13]. While the prospective evidence on the size of one’s SN on QoL is well-established, the role of other elements such as the diversity of SN appears weaker [14]. Although the mentioned evidence is mostly consistent, studies are mainly UK-based and may not be transferable to other regions. This is especially important considering that the life course patterns in QoL differ between regions [2]. In Central and Eastern Europe (CEE), one small longitudinal study suggested that Polish adults diagnosed with schizophrenia who had larger SN had higher QoL 7 years later [15], but no studies to date have assessed the prospective relationship between aspects of the SN and QoL in CEE using a population-based sample of older adults.

Furthermore, the role of depression in the relationship between SN and QoL at older ages may be especially important in CEE. While the evidence linking lack of social contacts with the onset of depression is not entirely consistent [16–18], a systematic review examining the longitudinal associations between social relationships and late-life depression suggested that fewer social interactions strongly predicted depressive symptoms [19]. The strength of this association was greater in Eastern Europe than in Western Europe [19]. Depression, in turn, has been identified as an important determinant of QoL at older ages. A systematic review found a significant inverse association between higher depressive symptoms and lower levels of QoL [20], although only two of the 74 reviewed studies used validated measures of eudemonic wellbeing [20]. Nonetheless, longitudinal evidence that explicitly measured QoL as a dimension of eudemonic wellbeing found that apart from baseline QoL and age, depressive symptoms were the strongest predictors of age-related changes in QoL [12]. Although it is theoretically plausible to surmise that depressive symptoms may mediate the relationship between SN and QoL, this mediational hypothesis has not been formally examined in older adults from CEE.

Therefore, this study aimed to assess the prospective relationship between frequency of contact with friends and relatives and QoL as a proxy for eudemonic wellbeing in an older CEE population; and to evaluate whether depressive symptoms mediated the hypothesised longitudinal relationship.

## Methods

### Study population

The Health, Alcohol and Psychosocial factors in Eastern Europe is a population-based cohort study conducted in the Czech Republic (six towns), Poland (Krakow), Russia (Novosibirsk) and Lithuania (Kaunas). As Lithuania joined the project at wave two, we used Czech, Polish and Russian participant data from wave 1 (2002–2005) and wave 2 (2006–2008) for the analyses. Participants aged 45–69 were recruited at baseline from the population registers of the countries’ towns. The wave one response rate varied from 55 to 61% [21].

### Quality of life

QoL was measured using the CASP (Control, Autonomy, Self-realisation and Pleasure) scale. CASP is a validated self-administered instrument used as a measure of eudemonic wellbeing in older populations based on the measurement of those four domains [22]. CASP asks participants to rate selected items on a Likert scale ranging from 0 (‘never’) to 3 (‘often’). QoL was measured by the 19-item version at baseline and the CASP-12 v.1 at follow-up developed by Borsch-Supan et al. [23]. Using wave 1 data from the twelve CASP-19 items comprising CASP-12 and CASP-12, v.1 scores were calculated by adding these item responses to derive scores ranging from 0 to 36 at both waves. Higher scores represent higher QoL.

### Social networks

Data on SN were collected at wave 1. Participants were asked ‘are you regularly in contact with your relatives who do not live in your household?’ and ‘how often do you visit friends?’ Responses ranged from 0 = ‘No relatives/No friends’, 1 = ‘Less than once a month’, 2 = ‘About once a month’, 3 = ‘Several times a month’, 4 = ‘About once a week’ to 5 = ‘Several times a week’. Responses to both questions were summed to create a score ranging from 0 to 10, where higher scores indicated more frequent contacts with both friends and relatives. Data on SN were analysed as scores, as well as tertiles denoting low (0–4), medium (5–6) and high (7–10) groups to capture both the change by unit and the gradient of change.

### Depressive symptoms

Depressive symptoms were measured using the Centre for Epidemiologic Studies Depression (CES-D) scale at waves 1 and 2, using the 20 and 10-item versions, respectively. CES-D has been shown to sufficiently screen for depression in older populations [24]. The CES-D 20 evaluated the frequency of 20 depressive symptoms during the past week on a four-point scale ranging from 0 (‘less than one a day’) to 3 (‘5–7 days’). Responses were added to obtain CES-D 20 scores (0–60). CES-D 10 asked whether 10 symptoms were felt ‘for much of the time’ in the previous week using yes (1) or no (0) response options, then summed to derive scores (0–10). Using suggested cut-offs, participants with CES-D 20 scores of ≥ 16 and CES-D 10 scores of ≥ 4 were identified as possible cases of depression.

### Covariates

Age, age squared, gender, country, marital status, educational attainment and material deprivation (based on how often participants lacked money for food, clothing or paying bills) were obtained at wave 1. Other baseline covariates included alcohol intake (grams per week), smoking status, self-rated health, physical functioning assessed with the SF-36 physical functioning subscale score [25] (0–100) and number of hours per week spent on doing sports or exercise.

### Statistical analyses

The CASP scale at wave 1 was included in the retirement module given to retired individuals who received a pension. Of the total baseline sample (*n* = 28,945), wave 1 CASP data were available for 12,840 participants. Of these participants, 39.79% (*n* = 4981) were excluded due to missing CASP data at wave 2. The analytic sample was further reduced by 0.72% (*n* = 57) and 21.74% (*n* = 1696) due to missing data on SN and covariates, respectively. Therefore, the analytic sample of complete cases was *N* = 6106.

Linear regression was used to assess the prospective association between frequency of contact with friends and relatives at baseline, measured in a linear fashion and in tertiles, and QoL at follow-up. The analyses fitted nested models which were adjusted for age, age squared, gender, country and marital status (model 1), socioeconomic and health-related covariates (model 2) and further adjusted for CASP-12 score at wave 1 (model 3). We also ran multivariable regressions to determine the explanatory role of each covariate included in model 2 and 3, which adjusted for each of these variables in turn. Interactions between the exposure and age, gender and country on QoL were tested using two-way interaction terms and likelihood ratio tests. As no interaction effects reached statistical significance, the models were not stratified by these covariates.

**Fig. 1:**
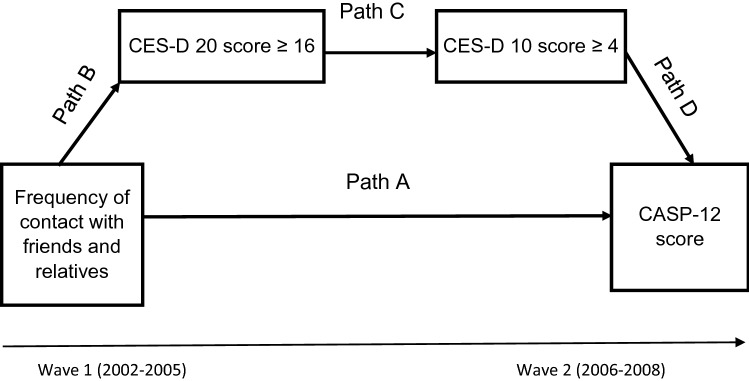
Path diagram of the association between frequency of contact with friends and relatives and QoL via depression

Path modelling was applied to test whether depressive symptoms at wave 1 and wave 2 mediated the longitudinal association between frequency of contact with friends and relatives at wave 1 and QoL at wave 2 [26]. The path model is based on a half-longitudinal design [27] because SN and depressive symptoms at wave 1 on one hand, and depressive symptoms and CASP-12 score at wave 2 on the other hand, were measured concurrently. The potential pathway via depression was hypothesised in a path model, which comprised four paths (Fig. 1). Path A, the ‘direct effect’ of frequency of contact with friends and relatives at wave 1 on QoL at wave 2, was measured using linear regression. Path B measured the effect of frequency of contact with friends and relatives on possible depression (both at wave 1) using logistic regression. Path C estimates the effect of being a possible case of depression at wave 1 on being a possible case of depression at wave 2, also using logistic regression. The last path, D, measured the effect of having possible depression on QoL (both at wave 2) using linear regression. Therefore, paths A and D are reported as linear regression coefficients, and paths B and C are reported as ORs. After estimating the path model, the indirect effect was calculated by multiplying the coefficients of paths B, C and D. The total effect was the sum of both the direct and indirect effects. The proportion of the total effect that was mediated through depressive symptoms was calculated by dividing the indirect effect by the total effect. The path model adjusted for the covariates used in the linear regression model by including them as predictors of QoL at wave 2 and frequency of contacts with friends and relatives at wave 1**.** For ease of interpretation, the path model was fitted using the continuous variable of frequency of contact with friends and relatives. All statistical analyses were carried out with Stata version 13 and with a 0.05 significance level.

### Sensitivity analyses

To assess possible biases in this study, several sensitivity analyses were carried out. To assess whether the analytic sample was representative of the recruited sample in HAPIEE, an analysis comparing the participants’ characteristics between the analytical sample (*n* = 6106) and those excluded (*n* = 6734) was done. Of the 12,840 participants with data on QoL at baseline, 7791 participants had available data on CASP-12 at wave 2 and on covariates included in model 1. Therefore, this model was re-run on these participants and the results were compared with those from the main analysis for robustness. Additionally, the cross-sectional association between frequency of contact with friends and relatives and QoL was assessed using the data available at baseline for CASP-12. As the main exposure in this study was derived from two variables, the prospective association was tested in each separate exposure, with a score ranging from 0–5 where a higher score indicates a more frequent contact. Lastly, to address the risk of over-adjustment by depression in the path analysis, another path model was estimated using only depression at wave 1.

## Results

### Description of the sample

Study characteristics of the sample differentiated by country can be seen in Table 1. The average age of the participants was 62.5 years. There were approximately 20% more women than men in the sample and around three-quarters of the sample lived with a partner at baseline. Overall, the mean CASP-12 score was 23.3 and 24.9, respectively, at baseline and follow-up. At both time points, Russian participants had the lowest mean score, while Czech participants had the highest. Frequency of contact with friends and relatives was higher among Czech participants, followed by Russian participants and, lastly, Polish participants, with a median of 7, 6 and 5, respectively. The interquartile range (IQR), indicating the variation of this variable, was the same for all countries. At baseline, a quarter of the total sample was classified as having probable depression. This proportion increased to 30.6% at follow-up.

**Table 1:** Study characteristics of the analytic sample by country

Variable	Czech republic *N* = 1596	Russia *N* = 1784	Poland *N* = 2726	Total *N* = 6106
Mean age, years (SD)	63.6 (4.4)	63.0 (5.1)	61.6 (5.6)	62.5 (5.2)
Female (%)	935 (58.6%)	1228 (68.8%)	1489 (54.6%)	3652 (59.8%)
Mean CASP-12 score at W1 (0–36) (SD)	23.3 (5.3)	20.7 (6.0)	24.9 (5.7)	23.3 (6.0)
Mean CASP-12 score at W2 (0–36) (SD)	27.4 (4.9)	21.3 (6.2)	25.8 (5.8)	24.9 (6.2)
Median frequency of contact with friends and relatives score (0–10) (IQR)	7 (5.8)	6 (4.7)	5 (3.6)	6 (4.7)
Frequency of contact with friends and relatives tertiles (%)				
Low (0–4)	16.5%	27.0%	42.9%	31.4%
Medium (5–6)	31.6%	37.3%	33.3%	34.0%
High (7–10)	51.9%	35.7%	23.8%	34.6%
CES-D 20 score ≥ 16 at W1 (%)	273 (17.1%)	544 (30.5%)	687 (25.2%)	1,504 (24.6)
CES-D 10 score ≥ 4 at W2 (%)	197 (12.3%)	832 (46.6%)	840 (30.8%)	1869 (30.6%)
Not partnered (%)	385 (24.1%)	591 (33.1%)	696 (25.5%)	1672 (27.4%)
Educational attainment (%)				
Primary	204 (12.8%)	160 (9.0%)	84 (14.1%)	784 (12.3%)
Vocational	600 (37.6%)	475 (26.6%)	634 (23.3%)	1,709 (28.0%)
Secondary	606 (38.0%)	704 (39.5%)	1,115 (40.9%)	2,425 (39.7%)
University	186 (11.6%)	445 (24.9%)	593 (21.8%)	1,224 (20.1%)
Smoking status (%)				
Smoker	281 (17.6%)	277 (15.5%)	656 (24.1%)	1,241 (19.9%)
Ex-smoker	511 (32.0%)	227 (12.7%)	831 (30.5%)	1,569 (25.7%)
Never smoker	804 (50.4%)	1280 (71.8%)	1239 (45.5%)	3323 (54.4%)
Median alcohol intake (grams per week) (IQR)	40 (0.120)	0 (0.0)	0 (0.20)	0 (0.40)
Self-rated health (%)				
Very good/good	564 (35.3%)	76 (4.3%)	692 (25.4%)	1332 (21.8%)
Average	855 (53.6%)	1150 (64.5%)	1557 (57.1%)	3562 (58.3%)
Poor/very poor	177 (11.1%)	558 (31.3%)	477 (17.5%)	1,212 (19.9%)
Median physical functioning (0–100) (IQR)	85 (75.95)	80 (60.95)	80 (65.95)	85 (65.95)
Median hours of weekly physical activity (IQR)	3 (0.8)	0 (0.3)	5 (0.10)	3 (0.7)
Median deprivation scale (0–12) (IQR)	0 (0.2)	4 (1.6)	1 (0.4)	2 (0.4)

### Prospective linear regression results

Table 2 reports the prospective association between frequency of contact with friends and relatives at baseline and CASP-12 score at follow-up. For every one unit increase in contact frequency score, there was a 0.31 point (95% CI 0.24, 0.37) increase in CASP-12 score, after controlling for demographic factors (Model 1). According to tertiles of contact frequency, people in the medium and high tertiles had a higher CASP-12 score by 1.12 (95% CI 0.76, 1.47) and 1.53 (95% CI 1.17, 1.90) units, compared to those in the low tertile. The addition of socioeconomic and health-related covariates in the model reduced these coefficients, but they remained statistically significant (Model 2). For every one unit increase in contact frequency score, CASP-12 score increased by 0.21 (95% CI 0.15, 0.27) points. CASP-12 scores for participants in the medium and high tertiles of frequency of contact with friends and relatives remained higher by 0.77 (95% CI 0.44, 1.10) and 1.01 (95% CI 0.67, 1.36) units, respectively, compared to the reference group. Finally, the inclusion of baseline CASP-12 in the model further attenuated the prospective associations (Model 3), as every one unit increase in frequency of contact score was significantly associated with a smaller increase of 0.12 (95% CI 0.06, 0.17) in CASP-12 score at follow-up. The fully adjusted results, however, showed a strong positive association as CASP-12 scores for participants in the medium and high groups were greater by 0.50 (95% CI 0.19, 0.82) and 0.59 (95% CI 0.26, 0.92) units, compared to the reference group.

**Table 2:** Regression coefficients (95% CI) for CASP-12 scores at follow-up by frequency of contact with friends and relatives at baseline

	Model 1			Model 2			Model 3		
	Coefficient	95% CI	*p* value	Coefficient	95% CI	*p* value	Coefficient	95% CI	*p* value
Frequency of contact with friends and relatives score (0–10) (B)	0.31	0.24, 0.37	< 0.001	0.21	0.15, 0.27	< 0.001	0.12	0.06, 0.17	< 0.001
Low tertile	REF			REF			REF		
Medium tertile (OR)	1.12	0.76, 1.47	< 0.001	0.77	0.44, 1.10	< 0.001	0.50	0.19, 0.82	0.002
High tertile (OR)	1.53	1.17, 1.90	< 0.001	1.01	0.67, 1.36	< 0.001	0.59	0.26, 0.92	< 0.001

Model 1: adjusted by country, gender, age, age square, marital status. Model 2: adjusted model 1 covariates plus education, deprivation, *SRH* physical activity, smoking status, alcohol intake and physical function. Model 3: adjusted by model 2 covariates plus baseline CASP−12

The addition of covariates reduced the regression coefficient by approximately a third in Models 2 and 3, although the effect on frequency of contact with friends and relatives remained highly statistically significant (*p* > 0.001). Among the covariates included in Models 2 and 3, the largest reduction in effect size was due to baseline CASP-12 score (Supplementary Table 1) which by itself decreased the effect by a third; followed by self-rated health.

### Path model results

**Fig. 2:**
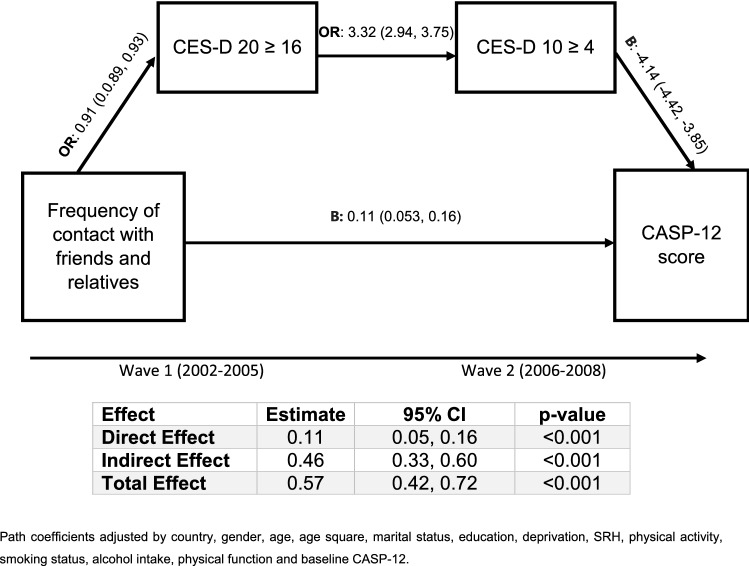
Path model coefficients and 95% CIs of the association between frequency of contact with friends and relatives and QoL via depression

Figure 2 displays the path model results. After accounting for all other paths in the model, the prospective effect of frequent contact with friends and relatives on QoL remained statistically significant. The direct effect showed that per every one unit increase in contact frequency score at baseline, the CASP-12 score increased by 0.11 (95% CI 0.053, 0.16) points at follow-up. The indirect paths were also statistically significant. For every one unit increase in contact frequency, the odds of being a possible case of depression at baseline were reduced by 9% (95% CI 0.89, 0.93). Subsequently, possible cases of depression at baseline were 3.32 (95% CI 2.94, 3.75) times more likely to be possible cases of depression at follow-up. Possible cases of depression at follow-up had, on average, a CASP-12 score that was 4.14 points lower (95% CI − 4.42, − 3.85) compared to non-cases at follow-up, accounting for the other paths and covariates in the model.

The total effect of a unit increase in frequency of contact with friends and relatives on CASP-12 score at follow-up was 0.57 (95% CI 0.42, 0.72). The indirect effect, via depression was calculated at 0.46 (95% CI 0.33, 0.60), increases the CASP-12 score at follow-up (per every one unit on exposure), suggesting that approximately 80% of the total effect was mediated through possible depression at baseline and follow-up.

### Sensitivity analyses

Participants who were excluded from the analysis due to missing data on baseline covariates and QoL at follow-up showed some significant difference from the analytical sample (Supplementary Table 2). Excluded individuals tended to have less frequent contacts with friends and relatives and were more likely to have possible depression at both time points compared to the analytical sample. Excluded individuals also had a slightly lower QoL score at both baseline and follow-up, alongside lower levels of educational attainment and less time spent on physical activity and lower proportion of smokers.

Repeating the linear regression model 1 on a larger share of individuals with complete information on the model 1 covariates generated results that were consistent with the main model 1 results (Supplementary Table 3). We estimated the cross-sectional association using nested linear regression models on all participants with complete data on baseline QoL and covariates. While the cross-sectional effect sizes were slightly larger than the prospective effect sizes in the main analyses, the difference in magnitude was not large enough to indicate that the prospective association was appreciably underestimated (Supplementary Table 4). Additional prospective analyses using separate contact frequency scores for friends and relatives indicated that both contact with friends and contact with relatives have independent but similar beneficial effects on QoL (Supplementary Table 5).

Finally, the path analysis considering only possible cases of depression at baseline showed a smaller indirect effect compared to the analysis using possible depression at both waves. The indirect effect of baseline depression, however, was strong and significant at 0.20 (95% CI 0.11, 0.29), and attributed to 44% of the model’s total effect (0.45 95% CI 0.29, 0.61) (Supplementary Fig. 1 ).

## Discussion

The prospective findings of this study found that more frequent contacts with friends and relatives significantly predicted higher QoL in older adults from the Czech Republic, Poland and Russia. Moreover, it appeared that depressive symptoms play a relevant role in this relationship.

Although the nature of the hypothesised relationship between SN and QoL has not been investigated in population-based samples of older adults from CEE, findings were consistent with UK-based evidence. The magnitude of the effect of contact frequency with friends and relatives on QoL in our sample was remarkably similar to those observed for older English adults [12, 14].

Despite the fact that depressive symptoms have not been specifically examined as a mechanism for the relationship between SN and QoL, our findings are congruent with research in the area. There is evidence of depressive symptoms mediating the relationship between social support and health-related QoL [28, 29]. Similar methods were used to assess the mediating role of depressive symptoms in these studies, even when exposure and outcomes were different from this study. Longitudinal studies focusing on eudemonic wellbeing have shown an association with depression, where greater symptoms were associated with diminished QoL [12]. As depressive symptoms were assessed as a covariate and not a mediator in the mentioned study**,** the comparability of the results is limited. Nevertheless, the evidence is consistent regarding the negative association between depressive symptoms and QoL and is compatible with the hypothesised pathway effects in this study.

Apart from depressive symptoms, the available literature suggests other important mechanisms as to why older adults with more frequent interactions with friends and relatives tend to have improved QoL. Frequency of interactions, a structural aspect of SN, have been associated with Cohen’s and Wills’ main hypothesis [30]. This hypothesis posits that being embedded in SN provides a positive effect on the individual. It has been suggested that these structural aspects can enhance QoL [31]. The independent association of frequency of contact with QoL of this study, seen in both the regression and path analyses, fits with the mentioned hypothesis. However, it may not be the only mechanism that could explain this relationship.

Another plausible explanation is that people who have more frequent contacts can rely on higher levels of social support from their network. Face-to-face social contact is significantly related to adequate levels of perceived instrumental and emotional social support [32]. In turn, higher perceived social support is related to higher levels of QoL [11]. Functional aspects of SN such as social support, have been associated with Cohen’s and Wills’ buffer hypothesis [33]. Structural aspects of SN like frequency of contact can influence functional aspects of them, related to social support, which can ultimately have an independent effect on QoL. Extensive evidence has suggested a positive and perhaps bidirectional association between social support and depressive symptomology [19]. In this study, as the direct effect from SN and QoL remained strong after accounting for depressive symptoms, social support may also be an important explanatory mechanism for the strong associations observed in this population. A social support pathway was not incorporated into the path model because variables regarding social support were not available at the time.

This study encompassed some limitations. A potential source of information bias may arise from the measurement of variables. Although there is evidence of CASP-12v.3 being a better fit for this population [34], it could not be used due to lack of the variables for that version at baseline and follow-up in the HAPIEE study. Moreover, while contact frequency with friends and relatives has been operationalised as a marker of one’s SN [14], this measurement did not originate from a validated instrument. Another limitation of this study is the similarity between some items in the CASP-12 and CES-D scales, which could become a potential source of over-adjustment. For example, responses to the CASP-12-item scale, ‘I feel full of energy these days’ were very likely to coincide with those given to the CES-D 20 and 10 items, ‘I feel that everything that I did was an effort’. While the items were designed to capture self-realisation and somatic symptoms in CASP and the CES-D scales, in turn, there appeared to be an overlap between these two items in our data. However, the sensitivity analysis of the main path model also found that the share of the total effect due to depressive symptoms at baseline was considerable at 44%.

A recurring limitation of longitudinal studies is attrition [35]. As the analyses required data from participants who took part in both waves, this made selection bias more likely in our sample. From the total of individuals with data on QoL at baseline, about half of them had at least one missing variable. Although the analytical sample was sufficiently large, there was some evidence of less favourable characteristics among the excluded individuals compared to the analytical sample. These differences, although statistically significant, were not large in magnitude. A potential imputation of missing data was considered, however, it was decided not to perform it as both longitudinal and cross-sectional results have high levels of agreement, as well as the sensitivity analyses of excluded individuals (Supplementary Tables 2, 3 and 4). Due to these reasons, imputation would have not changed our results. Attrition also could have played a role in the increase of CASP-12 score at wave 2 compared to the baseline. Although the analysis was made on complete cases, if attrition was more likely to occur on less healthy individuals, the healthier individuals left could have increased their QoL at follow-up, given that there are theories that support an increase of QoL over time at old age. The socioemotional selectivity theory [36] posits that people draw more positive elements from their relationships as they get older, which could also explain higher levels of QoL at follow-up compared to baseline levels. Non-random attrition could undermine the generalisability of the results. The survival effect also needs due consideration. People with poor health and QoL are more likely to die [3, 37], leaving healthier people who might be more prone to increase in their QoL at follow-up, as explained by the socioemotional selectivity theory.

There are aspects of the path analysis that also need to be addressed. Due to the temporality of the measurements and the number of points in time where data was collected, it was only possible to construct a half-longitudinal design [38]. This type of path analysis is limited by the concurrent measurement of both exposure and mediator, which may lead to bias estimates. To reduce the potential bias of concurrent measurements, the sensitivity analysis (Supplementary Fig. 1) using only depressive symptoms at wave 1, to avoid measurement concurrency of the mediator and the outcome, was carried out. In this latter analysis, the indirect effect of depressive symptoms on QoL, although smaller, remained strongly significant. Still, the indirect effect may not be correct due to a model´s misspecification. Depressed individuals tend to engage less frequently with their SN as a result of their condition [39]. Given that one diagnostic criterion for depression is impairment of social functioning [40], a path model that hypothesises a relationship between depressive symptoms and QoL that is mediated by contact frequency is also conceivable. This possibility was deemed as unlikely due to the substantial evidence discussed earlier reporting associations and models in a similar fashion as our path model [19, 20, 28, 29]. The assumption of stationarity, a stable degree of change over time, cannot be tested with only two points of measurements. Although the half-longitudinal designs have limitations, adjustments for baseline elements in both the mediator and outcome were considered to reduce these limitations [27]. As the relationships between SN, depressive symptoms and QoL are complex, future research on older CEE adults should be replicated using data with a longer follow-up time where these temporal sequences can be evaluated.

Finally, there are other aspects of SN that are related to QoL that were not measured. Structural elements such as size of the SN and diversity need to be considered for a comprehensive assessment of the role of aspects of SN on QoL. Since there is evidence that these other aspects are especially important at older ages [8–10], it is feasible that our findings may not have fully captured the relationship between SN and QoL for older adults in our sample. Similarly, non-structural elements, such as quality of SN, have been posited to have a large influence on QoL [13], although for older population there is evidence of a weaker association compared to younger population [18]. However, they represent a key feature of SN and should be considered in further research. Although our exposure was a combination of two elements, this decision was made based on the literature and psychometric properties of the combined exposure. Moreover, a separate analysis of the exposures was made (Supplementary Table 5) and it showed significance for both exposures. Also, the possibility that residual confounding influenced our results cannot be discarded.

There are some strengths of the study that should be considered. The longitudinal design enabled us to provide the first report on the nature of this prospective relationship for older adults in CEE. The large sample size provided enough power to detect small effects in the examined relationships. Furthermore, QoL was measured using the CASP-12v.1, which is an instrument validated to measure eudemonic wellbeing in older populations. In terms of methods, the path analysis is an appropriate method to approach our mediational hypothesis.

Findings suggest that there is an opportunity to increase QoL with strategies that promote social engagement and participation in ageing populations from CEE. This study, therefore, draws attention to particular strategies and programmes to promote mental health and wellbeing in later life. Although individual-level interventions remain important, our study suggests that interventions should have a comprehensive approach that is embedded within the interpersonal and community spheres of older adults’ lives [10].

## Electronic supplementary material

Below is the link to the electronic supplementary material.

Supplementary file1 (DOCX 156 kb)
